# DropLab: an automated magnetic digital microfluidic platform for sample-to-answer point-of-care testing—development and application to quantitative immunodiagnostics

**DOI:** 10.1038/s41378-022-00475-y

**Published:** 2023-01-11

**Authors:** Xuyang Hu, Xiangyu Gao, Songlin Chen, Jinhong Guo, Yi Zhang

**Affiliations:** 1 China-Singapore International Joint Research Institute, Guangzhou, China; 2 Guangzhou DropLab Scientific Co. Ltd., Guangzhou, China; 3 School of Mechanical and Aerospace Engineering, Nanyang Technological University, Singapore, Singapore; 4 DropLab Scientific (Singapore) Pvt. Ltd., Singapore, Singapore; 5 School of Sensing Science and Engineering, Shanghai Jiao Tong University, Shanghai, China; 6 School of Electronic Science and Engineering, University of Electronic Science and Technology of China, Chengdu, China

**Keywords:** Microfluidics, Biosensors, Electrical and electronic engineering

## Abstract

In point-of-care testing (POCT), tests are performed near patients and results are given rapidly for timely clinical decisions. Immunodiagnostic assays are one of the most important analyses for detecting and quantifying protein-based biomarkers. However, existing POCT immunodiagnostics mainly rely on the lateral flow assay (LFA), which has limited sensitivity or quantification capability. Although other immunodiagnostic assays, such as enzyme-linked immunosorbent assays (ELISAs), offer more sensitive and quantitative results, they require complex liquid manipulations that are difficult to implement in POCT settings by conventional means. Here, we show the development of DropLab, an automated sample-in-answer-out POCT immunodiagnostic platform based on magnetic digital microfluidic (MDM) technology. DropLab performs microbead-based ELISA in droplets to offer more sensitive and quantitative testing results. The intricate liquid manipulations required for ELISA are accomplished by controlling droplets with magnetic microbeads using MDM technology, which enables us to achieve full automation and easy operations with DropLab. Four ELISAs (the sample in triplicates and a negative control) can be run in parallel on the thermoformed disposable chip, which greatly improves the throughput and accuracy compared to those of other POCT immunodiagnostic devices. DropLab was validated by measuring two protein targets and one antibody target. The testing results showed that the limit of detection (LOD) of DropLab matched that of the conventional ELISA in a microwell plate. DropLab brings MDM one step closer to being a viable medical technology that is ready for real-world POCT applications.

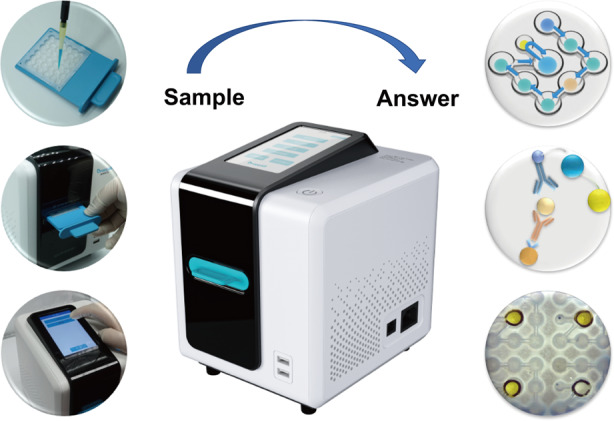

## Introduction

In vitro diagnostics (IVD) has attracted considerable interest because it facilitates clinical decision-making by analyzing easily accessible patient samples without the need for invasive medical procedures^1^. In contrast to centralized testing, where a large number of samples are collected and delivered to central laboratories for analysis, point-of-care testing (POCT) involves conducting IVD tests near patients at primary healthcare facilities, community pharmacies, physicians’ offices, or other decentralized sites^2^ with rapid turnaround time and reduced reagent cost and sample usage by using miniaturized testing instruments^2,3^, which are advantageous for diagnostics in resource-limited settings.

Immunoassays are one of the most important technologies for detecting and quantifying protein-based biomarkers^4^, and microfluidics is a promising technology to implement POCT immunoassays due to its portability, flexibility and cost-effectiveness^5–8^. For instance, paper-based microfluidic devices, such as lateral flow assays (LFAs), are widely used to test for pregnancy^9^, human immunodeficiency virus (HIV) infection^10,11^, and, more recently, COVID-19^12,13^. However, LFAs suffers from low accuracy^14–16^ and poor quantification capability and hence are used only for yes-no tests^17^. Other immunodiagnostic assays, such as enzyme-linked immunosorbent assays (ELISAs), offer more sensitive and quantitative results. However, they are often heterogeneous assays that require complex fluidic manipulations, which are difficult to implement in POCT settings. Although traditional closed-channel microfluidics devices can run ELISAs, intricate peripheral control systems are often needed, making them better suited to laboratories than POCT.

Magnetic digital microfluidics (MDM), which manipulates fluids in the form of droplets, is a preferred technology for POCT, especially for heterogeneous tests that require relatively complex liquid manipulation procedures and multiple separated reaction steps^2,18–20^. MDM is capable of the wide variety of droplet manipulations needed in most bioassays, such as droplet moving, droplet mixing, magnetic microbead extraction and droplet dispensing^21–24^, which facilitates the development of a fully functional microfluidic platform for POCT. For instance, an automated molecular testing platform based on MDM was used to detect hepatitis C virus and chlamydia using PCR^25,26^. Several MDM-based platforms were also demonstrated for ELISA^20,22,25^; however, none of them were equipped with both microfluidic manipulation and detection systems, nor could one of these platforms operate as a standalone device for POCT immunodiagnostics.

In this work, we present an automated MDM-based POCT platform with several innovative features that make it well suited for POCT immunodiagnostics. This MDM platform, known as DropLab, integrates a magnetic droplet manipulation module, an imaging-based optical detection module, and a disposable DropLab chip that is capable of analyzing four samples in parallel. The manipulation module controls the motion of droplets with a predefined moving sequence by synchronously moving four magnets mounted together on a rack. The optical detection module identifies reaction droplets under transillumination and measures their intensity. Surface topographic features^24^ are thermoformed on the DropLab chip to facilitate magnetic droplet manipulation. The DropLab platform includes interactive software with a user-friendly interface and a one-size-fits-all adapter for effortless chip loading, which may relieve the anxiety of nonprofessionals associated with operating medical instruments in POCT settings. To validate DropLab, ELISA was also performed for the detection of C-reactive protein, troponin C, and human IgG against SARS-CoV-2 spike protein. The results indicated that the performance of DropLab is comparable to that of conventional ELISA in microwell plates. DropLab provides an innovative approach to POCT immunodiagnostics and brings MDM technology one step closer to a viable medical technology that is ready for real-world applications.

## Results and discussion

### Overview of DropLab

**Fig. 1:**
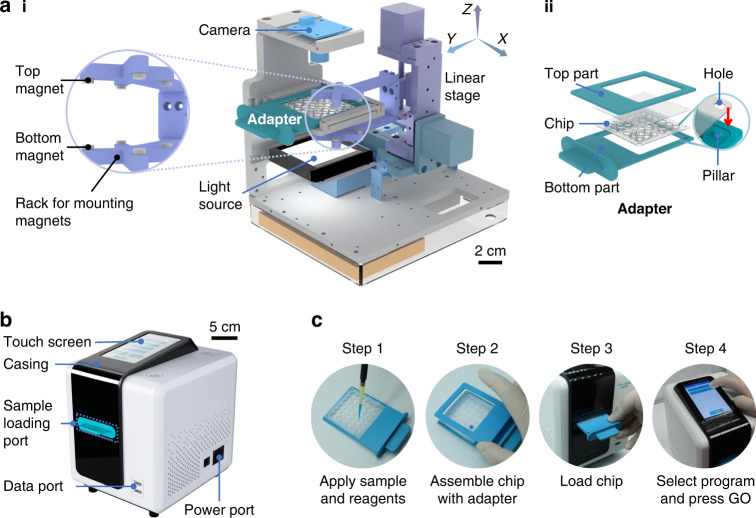
Overview of DropLab. **a** The internal mechanisms of the DropLab platform assembled view **ai**, including the magnetic droplet manipulation module, the optical module, and the one-fits-all adapter with the DropLab chip loaded **aii**. **b** Picture of the DropLab platform. **c** Workflow of performing POCT immunodiagnostics with DropLab

The proposed DropLab platform consists of a magnetic droplet manipulation module, an optical detection module, and a DropLab chip adapter (Fig. 1ai). Unlike most previously reported magnetic droplet manipulation platforms, which place only one magnet beneath the cartridge^20,22,24,27^, DropLab comprises two sets of magnets both beneath and above the DropLab chip. As shown in Fig. 1ai, the top and bottom magnets are mounted on a U-shaped rack to accomplish synchronous motion. Up to four sets of magnets can be mounted on the rack for the parallel processing of multiple samples in different units of the chip. The rack is mounted on a motorized 3-axis linear translational stage. The optical detection module includes a camera to capture the image of the droplets and a transilluminating light source that provides uniform illumination to the entire DropLab chip. DropLab chips with different internal designs can be loaded into the platform via the one-size-fits-all adapter (Supplementary Fig. S1). The small pillars on the bottom part of the adapter are locked to the positioning holes on the chip when both parts are pressed and secured in place by the adapter (Fig. 1aii). As shown in Fig. 1b, all the functional modules described above are integrated and housed in a casing (18 × 21 × 22 cm^3^) to prevent ambient light from interfering with image acquisition. The entire DropLab platform is powered by a 12 V/2 A power supply via the power port, and the test results can be transferred to a portable USB storage device via the data port. Supplementary Fig. S2 indicates the schematics of the control circuitry of DropLab. Figure 1c shows the workflow of immunodiagnostic tests on DropLab. All the required reagents are preloaded onto the chip. First, samples are applied to designated locations of the DropLab chip. After that, the chip is placed in the adapter and loaded into the platform. Then, users are prompted to select from a prestored list of programs via a touchscreen and press GO to initiate the test. The program includes a series of moving sequences that guide the magnet to automatically accomplish all the desired droplet manipulations for ELISA. The results will appear on the screen for user reference after the test is completed. Examples of the user interface of the interactive software and the prestored DropLab protocol for ELISA are presented in Supplementary Fig. S3 and Table S1.

### Design of the DropLab immunodiagnostic chip

**Fig. 2:**
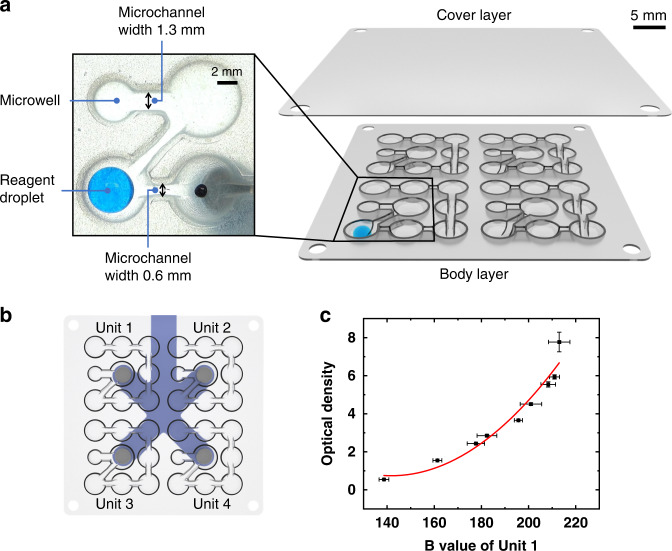
The DropLab chip. **a** Chip design with 4 individual units and a magnified image of the thermoformed microwells and microchannels. **b** The DropLab chip used in ELISA testing with 4 units for four parallel reactions. **c** The calibration curve of the image-based optical detection module that correlates OD with the droplet intensity

The DropLab chip shown in Fig. 2a consists of 4 parallel units (Fig. 2b, Units 1~4) that can be used to test the same sample in multiple replicates or test different samples on a single chip. The chip comprises a body layer and a cover layer. The body layer contains a series of internal assistive patterns, including microwells and microchannels (Fig. 2a), and is fabricated by thermoforming a thin thermoplastic polypropylene (PP) sheet with a thickness of 0.16 mm, which is a process compatible with large-scale manufacturing. The recessed microwells constrain the sample and reagent droplets to designated locations on the chip, and the microchannels connect the neighboring microwells and establish a path for the movement of droplets and microbeads. The size of each microwell and the width and depth of each microchannel are designed according to the droplet volume and desired droplet manipulations^24^. Figure 2a presents a magnified view of both wide (1.3 mm) and narrow (0.6 mm) microchannels. As our earlier work indicated^24^, a wider microchannel allows the droplet to pass through, and a narrow microchannel promotes the extraction of the microbeads from droplets. The cover layer serves two purposes. First, it provides a relatively enclosed space that prevents droplet evaporation. Second, the top surface acts as a substrate for the droplets and microbeads when the magnet approaches the chip from above. The body layer and the majority of the cover layer are spray-coated with a whitish superhydrophobic layer that facilitates the movement of droplets and microbeads. Specific regions of the cover layer are uncoated to prevent the whitish coating from blocking the view during image acquisition.

The optical detection module acquires images of droplets on the chip and analyses the results by extracting the intensity information of the reaction droplets. To correct for nonlinearity of the image-based intensity response to concentration, calibration curves are plotted by correlating the optical density (OD) of serial-diluted droplets measured by a UV‒Vis spectrophotometer with the inverse blue-channel intensity obtained from the droplet image. The calibration curve for Unit 1 is shown in Fig. 2c, and the other calibration curves are shown in Supplementary Fig. S4.

### Motion control of magnets

**Fig. 3:**
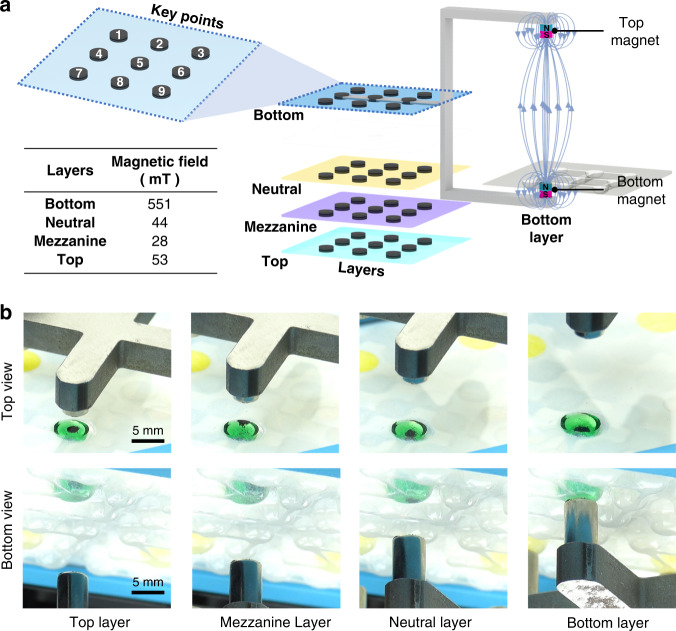
Schematic illustration of the motion control strategy for magnets. **a** The vertical Z direction is discretized into four layers (top, neutral, mezzanine and bottom), and 9 key points that correspond to the location of the microwells on the DropLab chip are defined in each layer (1–9). **b** Top view and bottom view of the chip and droplets when the magnet is in each of the 4 layers

As illustrated in Fig. 1a, the top and bottom magnets move synchronously together with the rack. The strength of the magnetic field and hence the behavior of the microbeads and droplets depend on the position and motion of the magnets. To simplify the setting of the motion sequence, the vertical Z direction is discretized into four layers (top, neutral, mezzanine and bottom), and nine key points that correspond to the location of the microwells on the DropLab chip are defined in each layer (1–9) (Fig. 3a). The magnet moves only among these predefined key points across the four layers, and each moving path is outlined by specifying the start and end points. For instance, the command “T1 to N5” means that the magnet moves from top layer point 1 to neutral layer point 5. As shown in Fig. 3b, the top magnet is in contact with the cover of the chip when it is in the top layer, where the magnetic field is 53 mT. In contrast, the bottom magnet is in contact with the bottom of the microwell of the chip when it is in the bottom layer, where the magnetic field is 551 mT. The magnetic field is generally weaker in other layers where the magnets have less effect on magnetic microbeads and droplets (Fig. 3a). In the top layer or bottom layer, the magnetic force is strong enough to concentrate all microbeads into a cluster either at the ceiling or the bottom of the microwell. In the mezzanine layer, the top magnet is able to partially attract microbeads so that the microbeads can move inside the droplet with the magnet but do not form a cluster. In the neutral layer, the magnetic microbeads are attracted to the bottom of the microwell by the bottom magnets; however, the force is not strong enough to cause the droplet to move.

**Fig. 4:**
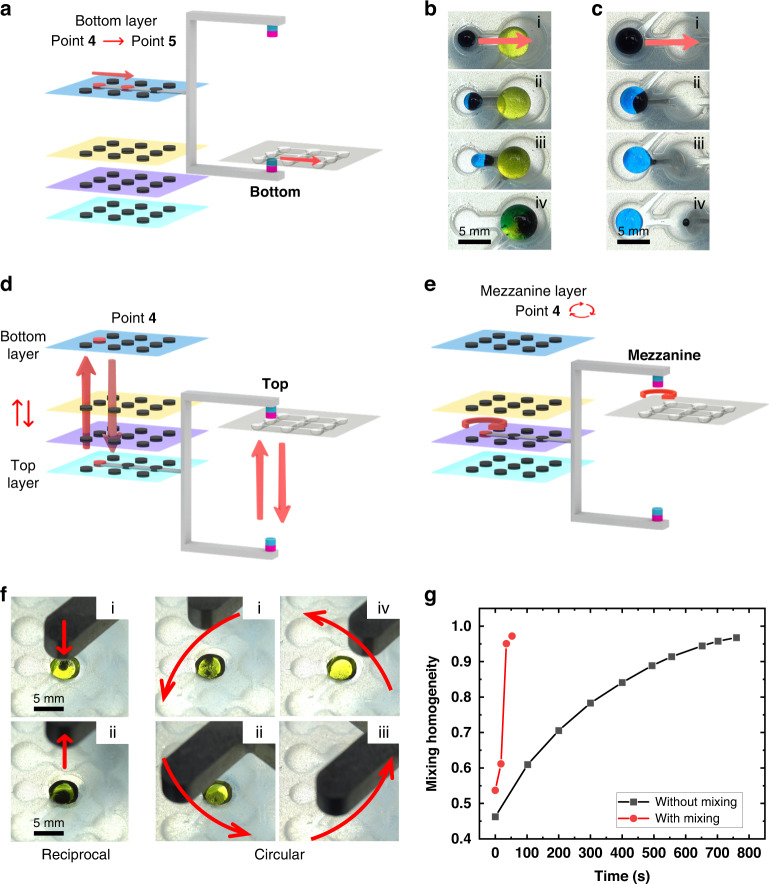
Droplet manipulation with DropLab. **a** Schematic illustration of the magnet motion required to perform droplet movement and microbead extraction. **b** Droplet movement and merging on DropLab. **c** Microbead extraction on DropLab. Schematic illustration of the **d** vertical reciprocal motion and **e** circular motion of the magnet required for mixing. **f** Droplet mixing on DropLab. **g** Change in droplet homogeneity over time with and without implementing the mixing strategy

Compared to the three-axis translational stage, it is more difficult to realize parallel droplet manipulation on the rotational motor-based platform^25^. For complex assays such as ELISA, nine reaction wells would take considerable space along a circular path. The only way to achieve parallel multiplexing is by arranging different units along the radial direction. However, the linear moving speed at different locations along the radial direction is different given the same angular velocity. As a result, the outer units and inner units may not undergo the same droplet operations. For instance, the moving speed in the outer unit may be high enough for bead extraction, but the speed may not be sufficient for the same droplet operation in the inner unit at a given angular velocity (Supplementary Fig. S5). Moreover, compared to other platforms that are capable of parallel processing, such as the IFAST platform^28^, DropLab is more flexible in terms of path planning. Other platforms are usually limited to a simple straight path, whereas DropLab can move in any arbitrary path defined by users according to the chip design. In addition, DropLab may move in several levels in the z direction to accomplish more complex fluidic operations, such as circular mixing, as demonstrated in Fig. 4.

### Droplet manipulation on DropLab

Previous studies have demonstrated a wide range of droplet manipulations on MDM platforms^19–22,24,27,29,30^, such as droplet movement, droplet splitting, microbead extraction, and cross-platform transfer. On DropLab, we mainly use droplet movement and microbead extraction to perform ELISA-based immunodiagnostic assays. The DropLab chip contains surface topographic features to assist microbead extraction.

Droplet movement is accomplished by moving the magnet between key points in the bottom layer (Fig. 4a). The magnet first moves in the neutral layer to the location of the magnetic microbeads. While moving in the neutral layer, the magnet has little effect on the droplet. Therefore, the neutral layer serves as a neutral position for homing and initiation. The magnets can safely move along the X and Y directions in the neutral layer without inducing undesired movement of droplets and magnetic microbeads. Next, the magnet moves to the bottom layer, and the bottom magnet touches the bottom of the microwell. When the bottom magnet approaches, the magnetic microbeads form a cluster. As the magnet moves from the current key point to the adjacent one, the magnetic microbeads move with it and drag the droplet along. The adjacent microwells are linked by a microchannel that acts as an assistive surface topographic feature. For a given amount of MagAttract Suspension G microbeads (~490 μg) and a droplet of a given size (10 μL, ~2.5 mm in diameter), if the microchannel is relatively wide (1.3 mm), the droplet can move through the microchannel to the adjacent microwell. If there is another droplet in the adjacent microwell, the two droplets can merge into one (Fig. 4b and Supplementary Video S1). In another scenario where the microchannel is relatively narrow (0.6 mm) compared to the size of the droplet (30 μL, ~3.7 mm in diameter), only the microbead cluster can move through the microchannel, whereas the droplet movement is constrained, resulting in the extraction of magnetic microbeads from the droplet (Fig. 4c and Supplementary Video S2).

A special magnet motion sequence is designed to enhance mixing in droplets and improve the quality of washing in ELISA. The proposed mixing strategy agitates microbeads in the droplet by moving the magnets both reciprocally in the vertical direction and circularly in the planar direction. During the vertical movement, the magnetic microbeads move up and down inside the droplet as the magnets move reciprocally between the top layer and bottom layer (Fig. 4d, f (reciprocal) and Supplementary Video S3). During planar motion, the magnet moves along a circular path at mezzanine layer (Fig. 4e, f (circular) and Supplementary Video S3), causing the microbeads to move about the droplet. The effect of the microbead motion is similar to that of a stirring pellet that agitates the fluid for efficient mixing. Each mixing cycle consists of 1 reciprocal vertical motion and 1 circular motion. To evaluate the effectiveness of the proposed mixing strategy, a yellow droplet (5 μL) was moved into a pure water droplet (50 μL), and a series of images of the merged droplet with and without implementing the mixing strategy were compared. Droplet homogeneity^22,30^, which was a measure of the variability of droplet pixel intensity, was used to evaluate the performance of the proposed mixing strategy in DropLab. As shown in Fig. 3g, the homogeneity of the droplet reaches a value >0.95 in ~54 s when implementing the mixing strategy (3 mixing cycles), whereas it takes ~700 s for the droplet to reach a similar level of homogeneity if the proposed mixing strategy is not used (where mixing occurs purely by diffusion).

### Immunodiagnostics on DropLab

ELISA is widely used for immunodiagnostics due to its high sensitivity and good quantification capability^31^. To validate DropLab for quantitative POCT immunodiagnostics, a total of three targets were tested, including two protein antigens, human C-reactive protein (CRP) and human troponin C protein (TnC), and one antibody, human IgG, against SARS-CoV-2 spike (S) protein. CRP is synthesized and released by the liver and is a well-established inflammatory marker widely used in the diagnostics of infections and many other diseases^32,33^. TnC is a subunit of the troponin complex and is a well-established biomarker for acute myocardial infarction (AMI)^34–36^. Human IgG against the S protein of SARS-CoV-2 is an indicator of immunity against COVID-19 as a result of past infection or vaccination and can serve as an “immune-triaging” marker^37^.

All three targets were tested with the same workflow (see Supplementary Table S1 for a detailed workflow protocol). The DropLab ELISA chip includes 4 units (Fig. 2b) that run 4 ELISAs in parallel, of which three units are used to analyze the sample in triplicate and the remaining unit is used as a negative control. Each unit contains nine microwells linked with eight microchannels. Microwells 1~7 (with a diameter of 6 mm), microwell 8 (with diameters of 7 mm) and microwell 9 (with a diameter of 4 mm) are designed to store reagents with volumes of 30, 50 and 10 μL, respectively. The reaction mixture, which contained 30 μL of sample, 5 μL of processed Dynabeads, and 0.5 μL of Qiagen magnetic microbeads, was added to microwell 1. Microwells 2~3 and microwells 5~7 were each loaded with 30 μL of washing buffer. Microwell 4 was loaded with 30 μL of detection antibody. Microwell 8 was loaded with 40 μL of 3,3’,5,5’-tetramethylbenzidine (TMB) enzyme substrate, and microwell 9 was loaded with 10 μL of stop solution.

**Fig. 5:**
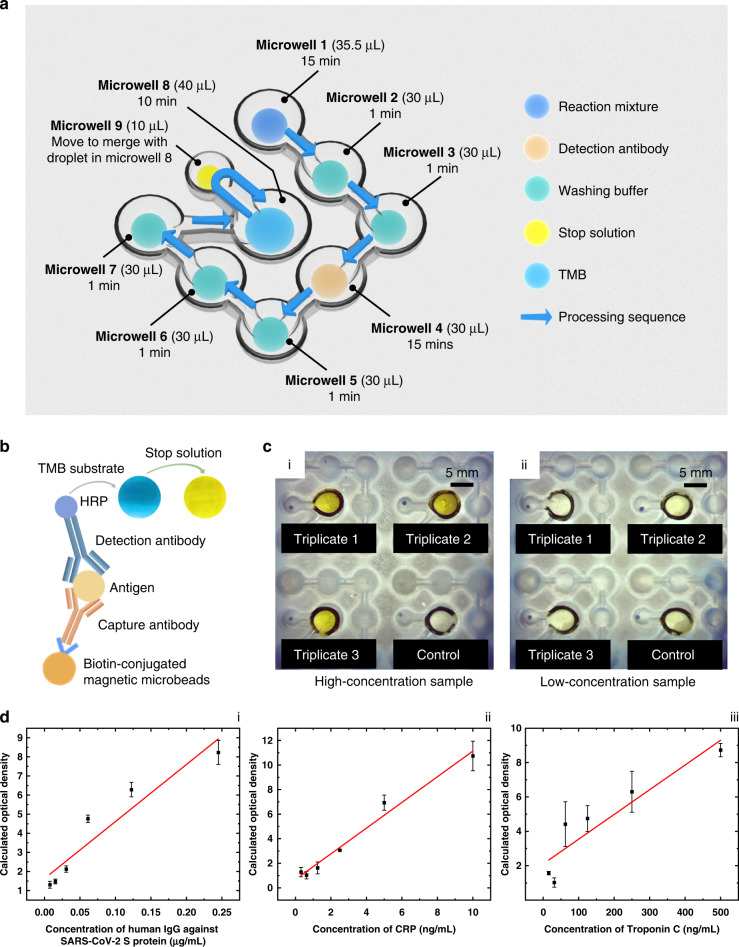
ELISA on DropLab. **a** Schematic illustration of droplets required for DropLab-based ELISA. **b** Schematic of microbead-based ELISA on DropLab. **c** Images of the CRP ELISA in a high sample concentration **ci** and a low concentration **cii**. **d** Standard curve of an ELISA for **di** human IgG against SARS-CoV-2 S protein, **dii** CRP and **diii** TnC

Taking antigen (CRP or TnC) detection as an example, the capture antibody was conjugated onto magnetic microbeads first, and the target protein was added to microwell 1 and incubated for ~15 min (Fig. 5a and Supplementary Video S4). During incubation, the droplet was mixed 50 times to fully expose the microbead surface to the aqueous solution, thereby promoting antigen-antibody binding on the microbead surface. After that, the magnet was moved to the bottom layer and held there for 20 s to attract the microbeads to the edge of the microwell. To wash away the waste, the microbeads were extracted from the reaction droplet and moved to the washing buffer droplet in microwell 2. The microbeads were incubated in the washing buffer solution for 1 min and mixed three times for thorough washing before being moved to microwell 3 for a second washing step. After washing, the microbeads with the target protein bound to the surface were moved to microwell 4 to merge with the droplet containing horseradish peroxidase (HRP)-conjugated detection antibody and incubated for 15 min with 50 cycles of mixing to promote antigen-antibody binding. After incubation, the capture antibody, target antigen and detector antibody formed a sandwich structure on the surface of the microbeads (Fig. 5b). Then, the microbeads were moved sequentially through microwells 5, 6 and 7 for three washes, each with three mixing cycles. Following these washing steps, the microbeads were moved to microwell 8 and incubated with the TMB droplet. Finally, the microbeads were extracted from the reaction droplet in microwell 8 and moved to microwell 9 to bring the stop solution droplet back to microwell 8 to merge with the reaction droplet. After the stop solution droplet was merged, the reaction droplets containing positive samples turned yellow, whereas the reaction droplets with negative controls remained relatively clear (Fig. 5c). Finally, an image of the entire chip was taken by a camera under uniform transillumination, and the intensity values from the R, G and B channels for all droplets were displayed on the screen. The overall sample-to-answer turnaround time was ~45 min. The retention rate^38^ was used to evaluate the losses of microbeads by using the workflow protocol. At a moving speed of 1 mm/s, ~78% of the microbeads remained in the final droplet on DropLab (see Supplementary Method). The retention rate is similar to those reported earlier^38^. Antibody detection was performed in a similar way except that the antigen (S protein) was conjugated to the microbeads as the capture agent, and human IgG against the S protein was the target.

A serial dilution of each target was measured on DropLab and benchmarked against a conventional ELISA performed in a microwell plate. The intensity value from the blue channel (B) of the droplet image was extracted and inverted (255-B). Samples with an inverse B value less than the threshold of the negative control (<140) were excluded from the analysis. In our case, only 1 datapoint for human IgG against SARS-CoV-2 S protein was excluded for this reason (see Supplementary Table S2 for details). Another datapoint for TnC was excluded because a surface defect caused microbeads to adhere to the surface (Supplementary Fig. S6). The inverse B value was then converted to OD using the calibration curve, and this calculated OD was plotted against the sample concentration to obtain the standard curve for each target. The standard curves were fitted with a linear regression model to determine the limit of detection (LOD)^39^. On DropLab, the LODs for human IgG against SARS-CoV-2 S protein, CRP and TnC were 0.091 μg/mL, 1.611 ng/mL and 235.561 ng/mL, respectively, which were similar to the LODs of ELISA in microwell plates for the tested concentration range (Supplementary Fig. S7 and Table S3).

The LOD for SARS-CoV-2 IgG is usually reported in terms of titers which cannot be directly translated to antibody concentration. The LOD of CRP is sufficient to differentiate normal and high levels during inflammation. The LOD for TnC is not sensitive enough for the diagnosis of myocardial infarction. However, the LOD of TnC on the benchmark microwell plate was also not sufficient. The current DropLab system does not emphasize the clinical relevance of the LOD because the purpose of the current study is to compare the performance of DropLab with benchmark ELISA in microwell plates. Therefore, ELISAs may be optimized by optimizing the antibody pair and assay conditions for clinical use in future work.

MDM provides a simple method of microfluidic manipulation, which enables us to achieve full automation and easy operations with DropLab. Furthermore, DropLab is lightweight, and the interactive software is user-friendly, the data transmission and device communication can be performed via Bluetooth and USB, and the thermoformed disposable chip is cost-effective. All these advantages substantially relive the user burden of operating a medical instrument POCT, which allows nonprofessionals to operate DropLab with minimal prior training. As a result, MDM is well suited for POCT immunodiagnostics, especially in primary healthcare settings. In POCT scenarios, one could either directly test whole blood samples^40,41^ by ELISA or use filter membranes or other cell separation devices suitable for POCT settings, such as a handheld centrifuge^42^, to obtain serum or plasma samples for testing with DropLab. In addition, DropLab runs triplicate samples and a negative control in parallel, which greatly improves the throughput and accuracy compared to other POCT immunodiagnostic devices. Although demonstrated only for immunodiagnostics with ELISA, DropLab is also compatible with molecular diagnostics and phenotypic analysis, as demonstrated on other MDM platforms^21,43^. DropLab chips of various designs could all fit into the adapter and be loaded onto the same DropLab platform for testing, although the optical detection system may require modification according to the sensing modality. Compared to these systems that are also based on immunoassays^44^, the main advantage of DropLab is the capability of multiplexing and parallel processing. Existing POCT platforms use cartridges for single samples. Due to the simple droplet-based fluidic operation, DropLab is able to integrate multiple reactions in parallel. Furthermore, both the material and manufacturing costs of the DropLab chip are expected to be lower than those of complex cartridges due to the simplicity of the DropLab system.

Despite these advantages, further improvements are still required before DropLab can be deployed for field testing. First, image-based detection has a narrow dynamic range. Under current conditions, DropLab can be used to analyze only samples whose concentrations range over roughly two orders of magnitude. A potential solution is to switch to chemiluminescent (CL) assays for immunodiagnostics and use more effective optical detectors, such as photomultiplier tubes or avalanche photodiodes, which may also significantly shorten the turnaround time. Another limitation is associated with reagent storage. Currently, all reagents are stored on chips in their liquid form at low temperature, and some are stored at fairly low concentrations. Such storage conditions are not suitable for long-term storage and are not preferred for POCT applications. To address this issue, we are currently exploring the storage of reagents on-chip in their dried form. We have made some progress and hope to report these developments in our next study. Last, DropLab is capable of sample-in-answer-out testing; however, the final results are reported only in terms of blue-channel intensity in the interactive software. Further analyses, such as quantification, are still performed off the platform. We are currently modifying the software to allow the input of user-specified calibration curves and standard curves for automated quantification.

## Conclusions

In summary, we have developed DropLab, an automated POCT platform based on MDM technology. It comprises a motorized motion control module for automated droplet manipulation, an image-based optical detection module for result acquisition, and a thermoformed disposable chip that is more cost-effective than conventional microfluidic chips. In addition, a one-size-fits-all adapter is able to accommodate various types of DropLab chips and expand the applicability to molecular diagnostics and phenotypical testing. In this study, we show that DropLab is able to automatically perform complex heterogeneous assays such as ELISA, which requires multiple stages of liquid and solid phase manipulations. Since it is fully automated and easy to operate, DropLab is well suited for POCT immunodiagnostics, especially in primary healthcare settings such as family clinics, the bedside and examination rooms. Compared to existing POCT immunodiagnostic devices such as LFAs, DropLab offers more sensitive and quantitative testing results that could match those of conventional ELISAs in microwell plates. Future work will focus on further improving the LOD and dynamic range of DropLab by incorporating more sensitive photodetectors and replacing ELISA with CL-based immunodiagnostic assays.

## Materials and methods

### Main components of DropLab

The DropLab manipulation module comprised three linear translational stages (DINGS’ Motion) that moved along the three principal axes. One cylindrical NdFeB (N52) magnet with a diameter of 5 mm and a height of 5 mm was mounted to the top of the U-shaped rack, and another NdFeB (N52) magnet with a diameter of 5 mm and a height of 10.5 mm was mounted to the bottom. All racks and coupling parts were fabricated by computer numerical control (CNC) machining.

A high-resolution camera (Sony) together with a rectangular light source (Dongguan CST) formed the optical detection module. The adapter was fabricated by stereolithography 3D printing (Creality). The outer casing (21 cm × 18 cm × 22 cm in length × width × height) was CNC machined. The touch screen (Shenzhen TXWEI) and graphic user interface (GUI) were designed to offer user-friendly operation and facilitate real-time observation of the internal conditions of the chip. The GUI and control software were programmed in Android Studio (Google Inc.). All systems in DropLab were controlled by a microcontroller (Shenzhen RERVISION).

### Design and fabrication of the DropLab chip

The body layer of the DropLab chip was fabricated by thermoforming a 0.16 mm-thick PP plastic sheet between a pair of customized aluminum metal molds at 90 °C and 0.5 MPa. The mold was designed using the computer-aided design software SolidWorks (Dassault Systèmes) and fabricated by CNC machining. The cover layer was made of a 0.16 mm-thick PP plastic sheet. The body layer and the cover layer were cut into a 60 mm by 60 mm square. The positioning holes on the body layer and the cover layer were created with a customized puncher.

The surface of the body layer and the cover layer was spray-coated with a superhydrophobic coating material (Shenzhen XINNA) to facilitate the movement of the droplets and magnetic microbeads. The superhydrophobic coating appeared whitish on the chip (see Supplementary Methods). To keep the observation areas (microwell 8) clear, four pieces of circular disks were taped to the cover layer as stencils. After spray coating, the stencils were removed, and the observation areas were hence not coated by the whitish coating material.

### Evaluation of mixing

To evaluate the effectiveness of the proposed mixing strategy, a 5 μL yellow droplet was moved into a 50 μL water droplet with magnetic microbeads. Pictures of the merged droplet at different time points after merging were taken by an external camera (Sony, DSC-RX10M4), and the pixel information of the droplet was extracted and analyzed with MATLAB (MathWorks).

The picture was first split into individual red, green and blue channels. The mixing index *M* of the blue channel was determined by calculating the standard deviation of the droplet pixel intensity according to Eq. 1^22,30^

1 $$M = \sqrt {\frac{1}{N}\mathop {\sum }\limits_{b = 1}^N \left( {I_b - \bar I} \right)^2}$$

where *M* is the mixing index, *N* is the total number of pixels of the droplet, *I*_*b*_ is the intensity of pixel b in the blue channel, and $\bar I$ is the average intensity of all droplet pixels in the blue channel.

The mixing homogeneity was calculated according to Eq. 2

2 $$H = 1 - \frac{{M_c - M_f}}{{M_i - M_f}}$$

where *H* is the mixing homogeneity, *M*_*c*_ is the mixing index at different time points after merging, and *M*_*i*_ and *M*_*f*_ are the initial and final mixing indices, respectively. The initial mixing index was that of a pure water droplet, and the final mixing index was that of a droplet containing water and yellow dye that was fully mixed by vortexing.

### Reagents and materials

For the detection of human IgG against SARS-CoV-2, biotinylated SARS-CoV-2 spike protein (40589-V27B-B, Sino Biological) was reconstituted with water to a concentration of 0.25 mg/mL and used as the capture agent for the detection of human IgG against SARS-CoV-2. HRP-conjugated rabbit anti-human IgG (ab6759, Abcam) was diluted to 20 ng/mL with phosphate-buffered saline (PBS) (SL6110, Coolaber) and used as the detector antibody.

To prepare the capture antibody for the detection of TnC, a biotin conjugation kit (ab201796, Abcam) was used for the biotin labeling of rabbit polyclonal anti-troponin c antibody (11011-T16, Sino Biological), which served as the capture antibody. The antibody concentration after labeling was 0.8 mg/mL. An HRP conjugation kit (ab102890, Abcam) was used for the HRP labeling of rabbit polyclonal anti-troponin c antibody that served as the detection antibody. The antibody concentration after labeling was 0.8 mg/ml, which was later diluted to 0.1 μg/mL with PBS for ELISA.

The capture antibody for ELISA detection was prepared by labeling mouse anti-human CRP antibody (SEK11250, 11250-MM07T, Sino Biological) with biotin using the biotin conjugation kit, and the antibody concentration after labeling was 0.8 mg/mL. HRP-conjugated rabbit anti-human C-reactive protein (SEK11250, 11250-R106, Sino Biological) was diluted to 0.1 μg/mL with PBS and used as the detector antibody.

The targets used in the ELISA experiments were prepared as follows. Human IgG against SARS-CoV-2 (AHA001, Sanyou Bio) was diluted to a concentration of 9.8 μg/mL with fetal bovine serum (FBS, FSP500, ExCell Bio) as a stock solution for serial dilutions. Human TnC protein (11011-HNAE, Sino Biological) was reconstituted in water to a concentration of 0.25 mg/mL and diluted to 1 μg/mL with FBS as a stock solution for serial dilution. Recombinant human CRP (SEK11250, Sino Biological) was diluted to a concentration of 10 μg/mL with FBS as a stock solution for serial dilution. The stop solution was 1 mol/L sulfuric acid, the HRP enzyme substrate was 3,3’,5,5’-tetramethylbenzidine (TMB) (34028, Thermo Fisher Scientific), and the washing solution was PBS with 0.05% Tween 20 (P1379, Sigma‒Aldrich).

### Preparation of magnetic microbeads

Streptavidin-coated Dynabeads (M-280 Streptavidin, Thermo Fisher Scientific) and MagAttract Suspension G (1026883, QIAGEN) were transferred to a microcentrifuge tube and washed twice with washing solution. To immobilize the SARS-CoV-2 spike protein, 10 μL of reconstituted biotinylated protein and 90 μL of carbonate buffer solution (CBS) were added to the microcentrifuge tube and incubated for two hours at room temperature. CBS was prepared by dissolving 3.03 g of sodium carbonate (S7795, Sigma‒Aldrich) and 6.0 g of sodium bicarbonate (S5761, Sigma‒Aldrich) in 1 L of water. The conjugated Dynabeads were washed twice with the washing solution after incubation, and the buffer was removed after the second wash. To immobilize human CRP and TnC, 0.625 μL of biotin-conjugated CRP capture antibody or 0.625 μL of TnC capture antibody was added to 100 μL of Dynabeads and incubated for two hours at room temperature. The Dynabeads were washed twice after incubation, and 100 μL of PBS was added after the second wash to resuspend the processed Dynabeads. MagAttract Suspension G (Qiagen) was used to enhance the magnetic response. All antibody-conjugated Dynabeads and MagAttract Suspension G were blocked with 100 μL of blocking buffer (37539, Thermo Fisher Scientific) for 1 h. They were washed twice with 100 μL of washing solution and resuspended in 100 μL of PBS.

### ELISA on DropLab

All the required reagents were preloaded into designated microwells of the DropLab chip, as shown in Fig. 5a. Five microliters of the capture antibody-conjugated Dynabeads and 0.5 μL of suspension G were loaded into microwell 1. Then, 30 μL of washing buffer was loaded into microwells 2, 3, 5, 6 and 7. Thirty microliters of detection antibody was loaded into microwell 4. Fifty microliters of TMB was loaded into microwell 8. Ten microliters of stop solution was loaded into microwell 9. The DropLab chip was sealed with a transparent film after reagent loading. The DropLab chip consists of 4 units that run four ELISAs in parallel (Fig. 2b). In our test, Units 1–3 were used to test each sample in triplicate, and Unit 4 was used as the negative control. To perform ELISA on DropLab, the sealing film was removed, and 30 μL of sample was added to microwell 1 of each unit. Then, the chip was placed into the adapter, and the assembled adapter was inserted into the sample loading port of DropLab (Fig. 1c). Programs comprising motion sequences required to manipulate microbeads and droplets for ELISA and other diagnostic assays were prestored in the DropLab platform. The ELISA program for ELISA is detailed in Supplementary Table S1. All users need to select the right program and press GO to initiate the test. The final results in terms of droplet intensity are displayed after the test (Fig. S3c). The acquired images could be exported to portable USB storage devices and transferred to other devices for further analysis.

### Data analysis

The software and GUI were based on the Android operating system and were developed using Android Studio (Google Inc.). The operation commands, including the number of mixing steps, starting and end points, regions of interest (ROIs), and other parameters (Supplementary Fig. S3), were preprogramed for each type of DropLab chip and stored in DropLab. Users may also define these parameters and program customized commands themselves. The pictures of the testing results were taken by the camera and stored as BMP files. The R, G and B values of the selected ROIs were displayed on the touchscreen after image acquisition. For validation of results in this study, the pictures were transferred and analyzed on a PC with ImageJ (National Institute of Health).

The concentration of the sample was reflected by the intensity of the droplet in the blue channel (B). The inverse blue intensity (255 - *B*) was converted to OD according to the calibration curve, which corrects for nonlinearity of the image-based intensity response to concentration. The calibration curve was obtained by correlating the OD and inverse blue intensity of developed TMB droplets of known concentrations. One calibration curve was created for each of the four units to account for possible nonuniform illumination. The OD values calculated based on the calibration curve were plotted against the concentration to obtain the standard curve.

All supplementary videos were taken in real time. The incubation step in Supplementary Video S4 was shortened so that we could demonstrate the entire immunodiagnostic ELISA process.

## Supplementary information

Supplemental Material

S1 droplet moving and merging on DropLab

S2 extraction

S3 mixing

S4 ELISA process
