# Supplementary material for: DropLab: an automated magnetic digital microfluidic platform for sample-to-answer point-of-care testing—development and application to quantitative immunodiagnostics
Source: Microsyst Nanoeng. 2023 Jan 11;9:10. doi: 10.1038/s41378-022-00475-y (PMC9833028; doi:10.1038/s41378-022-00475-y)
Supplement: Supplementary file 1 — Supplemental Material [file 41378_2022_475_MOESM1_ESM.docx]

**DropLab, An Automated Magnetic Digital Microfluidic Platform for Sample-to-Answer Point-of-Care Testing: Development and Application to Quantitative Immunodiagnostics**

Xuyang Hu^1,2^, Xiangyu Gao^1^, Songlin Chen^3,4^, Jinhong Guo^5*^, Yi Zhang^6^*

^1^China-Singapore International Joint Research Institute

^2^Guangzhou DropLab Scientific Co. Ltd.

^3^School of Mechanical and Aerospace Engineering, Nanyang Technological University

^4^DropLab Scientific (Singapore) Pvt. Ltd.

^5^School of Sensing Science and Engineering, Shanghai Jiao Tong University

^6^School of Electronic Science and Engineering, University of Electronic Science and Technology of China

Correspondence to Yi Zhang (yi_zhang@uestc.edu.cn) or Jinhong Guo (guojinhong@sjtu.edu.cn)


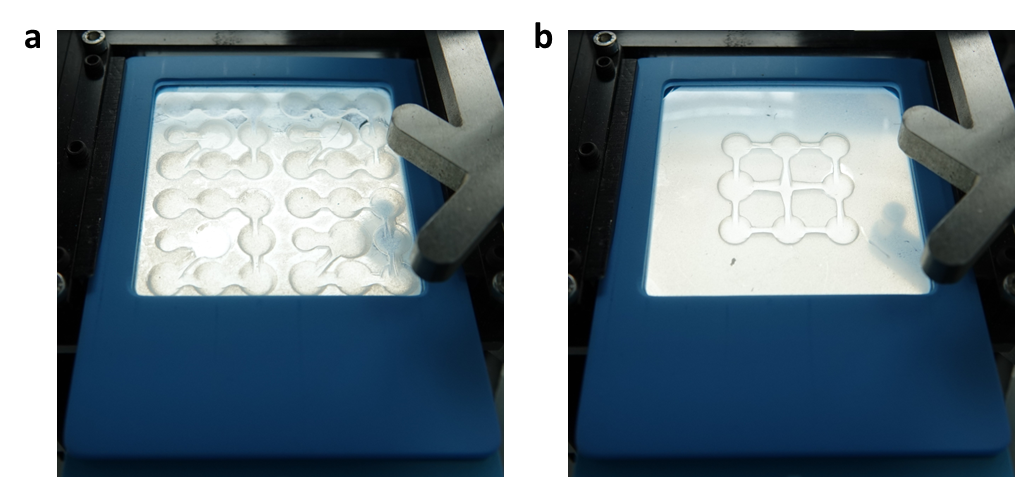


**Fig. S1: DropLab chips of different internal designs loaded into platform via the one-fits-all adapter**. **a** DropLab chip with 4 parallel units. b DropLab chip with 1 unit.


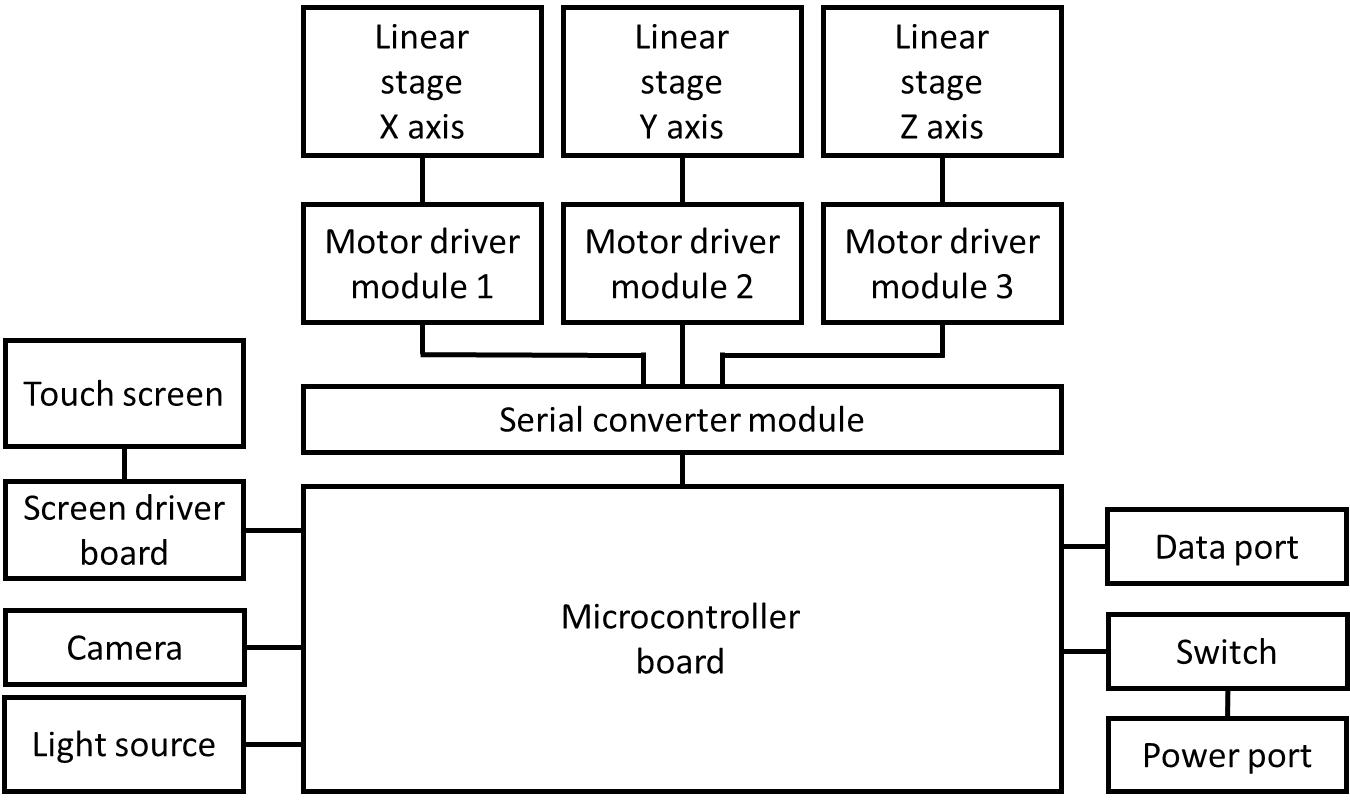


**Fig. S2: Schematic illustration of the control circuitry on DropLab**.


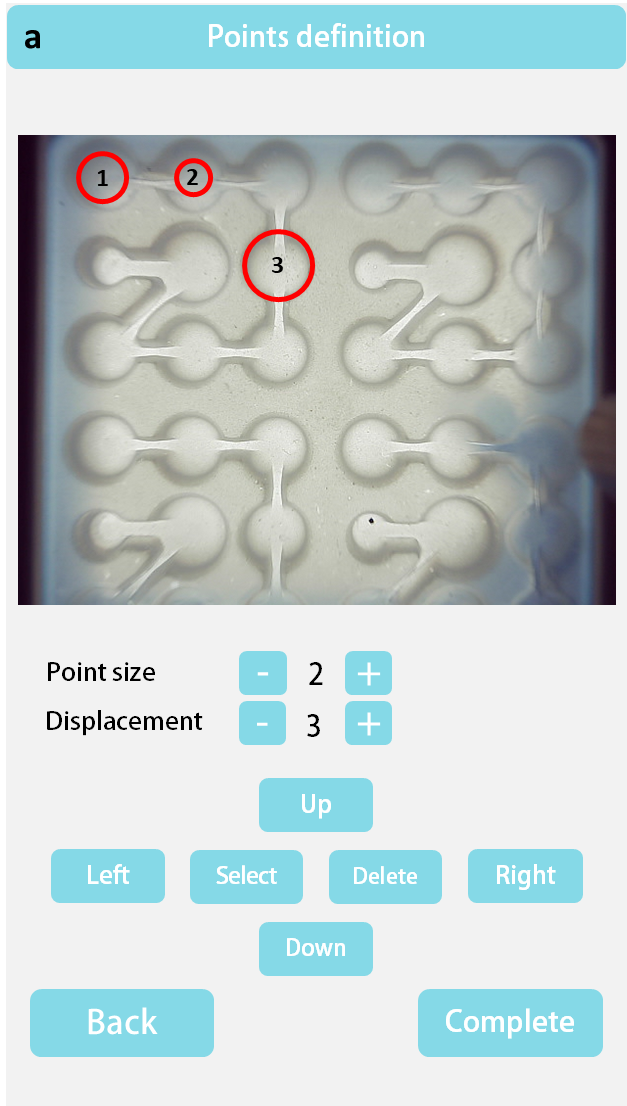

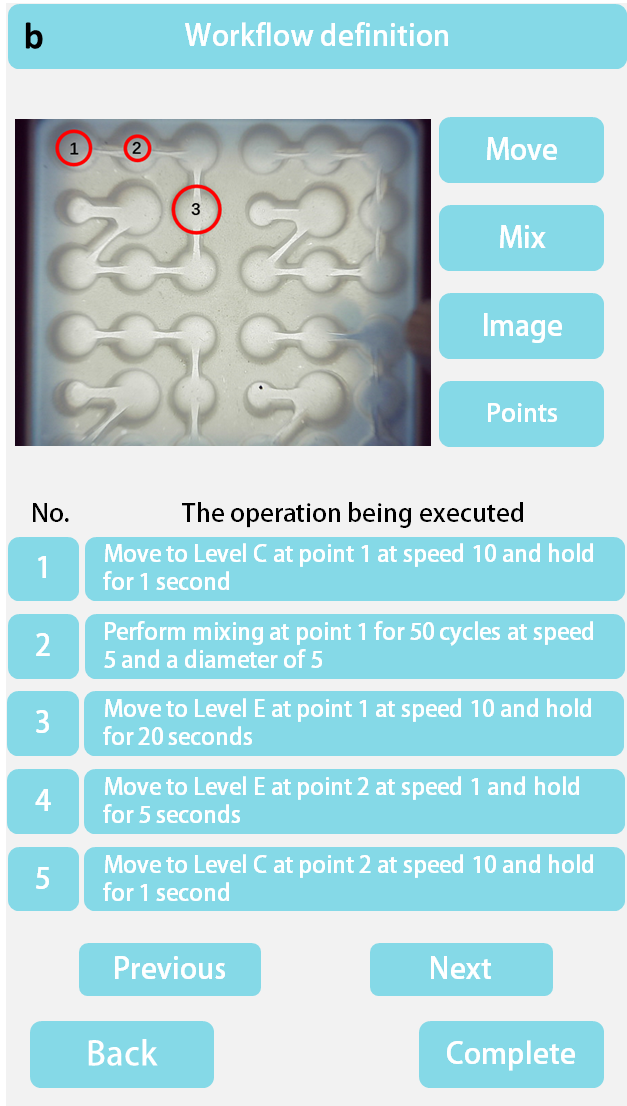

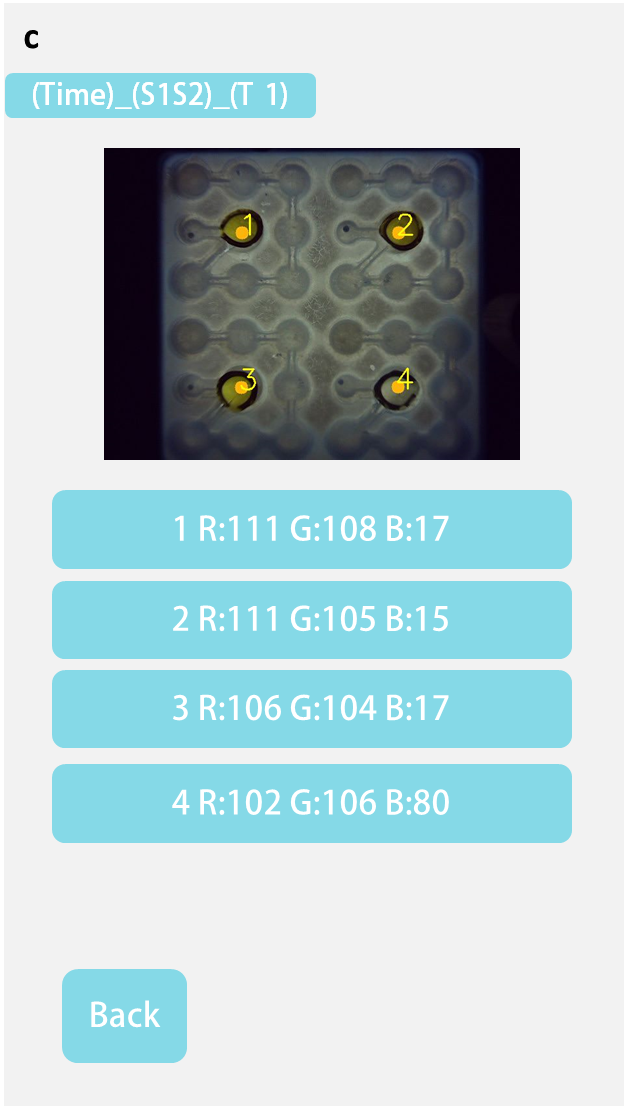


**Fig. S3: Representative user interface**. **a** The points definition function enables users to set the location of the control magnets in x-y plane **b** The workflow definition function enable users to define the movement of the control magnet. By choosing the “Move” icon, uses could set the point that being manipulated, the layers that the selected point can reach, the moving speed and the time that the control magnet suspended at this point. “Mix” enable users to define the repeat cycles of mixing manipulation, the diameter of the circular mixing, and the moving speed of the control magnet to execute the mixing commend. An image will be acquired as an “Image” command executed. “Points” allow users to chose and modify the selected points. **c** The result interface indicates the final image of the testing process and the reference color intensity information for the predefined ROIs.

**Tab. S1: DropLab protocol for ELISA**.

| **Sequence** | **Command** |
| --- | --- |
| **1** | Move to Neutral Layer at point 1 at speed 10 and hold for 1 second |
| **2** | Perform mixing at point 1 for 50 cycles at speed 5 and a diameter of 5 |
| **3** | Move to Bottom Layer at point 1 at speed 10 and hold for 20 seconds |
| **4** | Move to Bottom Layer at point 2 at speed 1 and hold for 5 seconds |
| **5** | Move to Neutral Layer at point 2 at speed 10 and hold for 1 second |
| **6** | Perform mixing at point 2 for 3 cycles at speed 5 and a diameter of 5 |
| **7** | Move to Bottom Layer at point 2 at speed 10 and hold for 20 seconds |
| **8** | Move to Bottom Layer at point 3 at speed 1 and hold for 5 seconds |
| **9** | Move to Neutral Layer at point 3 at speed 10 and hold for 1 second |
| **10** | Perform mixing at point 3 for 3 cycles at speed 5 and a diameter of 5 |
| **11** | Move to Bottom Layer at point 3 at speed 10 and hold for 20 seconds |
| **12** | Move to Bottom Layer at point 4 at speed 1 and hold for 5 seconds |
| **13** | Move to Neutral Layer at point 4 at speed 10 and hold for 1 second |
| **14** | Perform mixing at point 4 for 50 cycles at speed 5 and a diameter of 5 |
| **15** | Move to Bottom Layer at point 4 at speed 10 and hold for 20 seconds |
| **16** | Move to Bottom Layer at point 5 at speed 1 and hold for 5 seconds |
| **17** | Move to Neutral Layer at point 5 at speed 10 and hold for 1 second |
| **18** | Perform mixing at point 5 for 3 cycles at speed 5 and a diameter of 5 |
| **19** | Move to Bottom Layer at point 5 at speed 10 and hold for 20 seconds |
| **20** | Move to Bottom Layer at point 6 at speed 1 and hold for 5 seconds |
| **21** | Move to Neutral Layer at point 6 at speed 10 and hold for 1 second |
| **22** | Perform mixing at point 6 for 3 cycles at speed 5 and a diameter of 5 |
| **23** | Move to Bottom Layer at point 6 at speed 10 and hold for 20 seconds |
| **24** | Move to Bottom Layer at point 7 at speed 1 and hold for 5 seconds |
| **25** | Move to Neutral Layer at point 7 at speed 10 and hold for 1 second |
| **26** | Perform mixing at point 7 for 3 cycles at speed 5 and a diameter of 5 |
| **27** | Move to Bottom Layer at point 7 at speed 10 and hold for 20 seconds |
| **28** | Move to Bottom Layer at point 8 at speed 1 and hold for 5 seconds |
| **29** | Move to Neutral Layer at point 8 at speed 10 and hold for 1 second |
| **30** | Perform mixing at point 8 for 50 cycles at speed 5 and a diameter of 5 |
| **31** | Move to Bottom Layer at point 8 at speed 10 and hold for 20 seconds |
| **32** | Move to Bottom Layer at point 9 at speed 1 and hold for 5 seconds |
| **33** | Move to Neutral Layer at point 9 at speed 10 and hold for 1 second |
| **34** | Perform mixing at point 9 for 1 time at speed 5 and a diameter of 5 |
| **35** | Move to Bottom Layer at point 9 at speed 10 and hold for 20 seconds |
| **36** | Move to Bottom Layer at point 8 at speed 1 and hold for 5 seconds |
| **37** | Move to Neutral Layer at point 8 at speed 10 and hold for 1 second |
| **38** | Perform mixing at point 8 for 10 cycles at speed 5 and a diameter of 5 |
| **39** | Move to Bottom Layer at point 8 at speed 10 and hold for 20 seconds |
| **40** | Move to Bottom Layer at point 9 at speed 1 and hold for 5 seconds |
| **41** | Move to Neutral Layer at point 9 at speed 10 and hold for 1 second |
| **42** | Image acquisition |


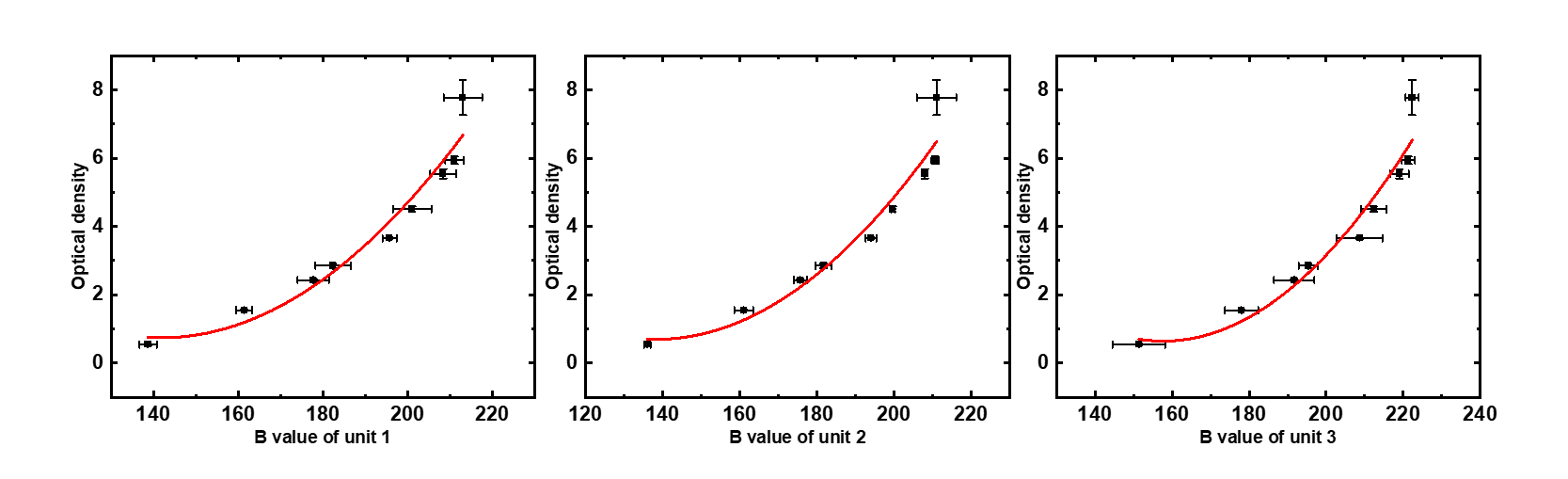


**Fig. S4: Calibration curve**.


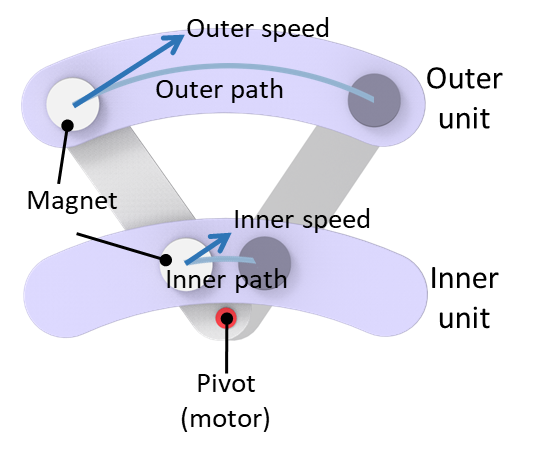


**Fig. S5: Motion illustration of a rotational motor-based platform.** Showing the differences of outer and inner unit in magnet speed and path for parallel assay.

**Tab. S2: Intensity readout and calculated OD of DropLab ELISA**.

| **Target** | **Conc.** | **Unit** | **Normalized Unit 1** | **Calculated OD 1** | **Normalized Unit 2** | **Calculated OD 2** | **Normalized Unit 3** | **Calculated OD 3** | **Valid No. of replicates** |
| --- | --- | --- | --- | --- | --- | --- | --- | --- | --- |
| Human IgG against SARS-CoV-2 S protein | 0.245 | μg/mL | 226 | 9.111 | 220 | 7.779 | 228 | 7.793 | 3 |
|  | 0.123 |  | 213 | 6.741 | 208 | 5.814 | 220 | 6.288 | 3 |
|  | 0.061 |  | 198 | 4.497 | 201 | 4.813 | 212 | 4.962 | 3 |
|  | 0.031 |  | 173 | 1.927 | 176 | 2.109 | 191 | 2.342 | 3 |
|  | 0.015 |  | 135 | 0.823 | 165 | 1.351 | 182 | 1.600 | 2 |
|  | 0.008 |  | 158 | 1.088 | 164 | 1.295 | 181 | 1.531 | 3 |
| CRP | 10.000 | ng/mL | 237 | 11.426 | 240 | 11.751 | 234 | 9.041 | 3 |
|  | 5.000 |  | 218 | 7.605 | 216 | 7.089 | 219 | 6.112 | 3 |
|  | 2.500 |  | 185 | 2.978 | 187 | 3.131 | 198 | 3.077 | 3 |
|  | 1.250 |  | 159 | 1.127 | 178 | 2.275 | 180 | 1.466 | 3 |
|  | 0.625 |  | 150 | 0.855 | 167 | 1.469 | 163 | 0.783 | 3 |
|  | 0.313 |  | 147 | 0.807 | 164 | 1.295 | 184 | 1.745 | 3 |
| Troponin C | 500.000 | ng/mL | 226 | 9.111 | 226 | 8.879 | 230 | 8.198 | 3 |
|  | 250.000 |  | 203 | 5.186 | 221 | 7.957 | 217 | 5.769 | 3 |
|  | 125.000 |  | 200 | 4.766 | 207 | 5.664 | 204 | 3.817 | 3 |
|  | 62.500 |  | 183 | 2.780 | 209 | 5.966 | 209 | 4.512 | 3 |
|  | 31.250 |  | 149 | 0.837 | 153 | 0.825 | 179 | 1.403 | 3 |
|  | 15.625 |  | 166 | 1.470 | 156 | 0.927 | 183 | 1.671 | 2 |


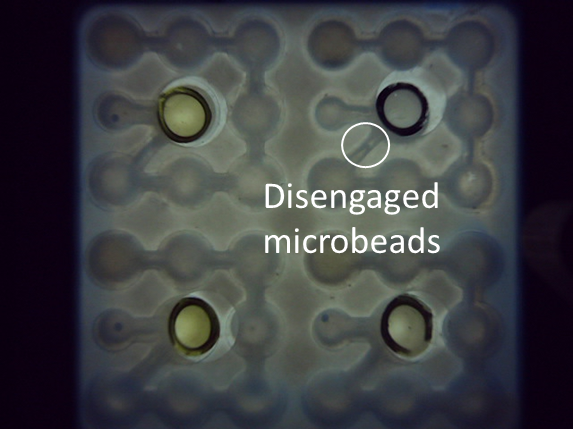


**Fig. S6: Failed test due to surface defect**.


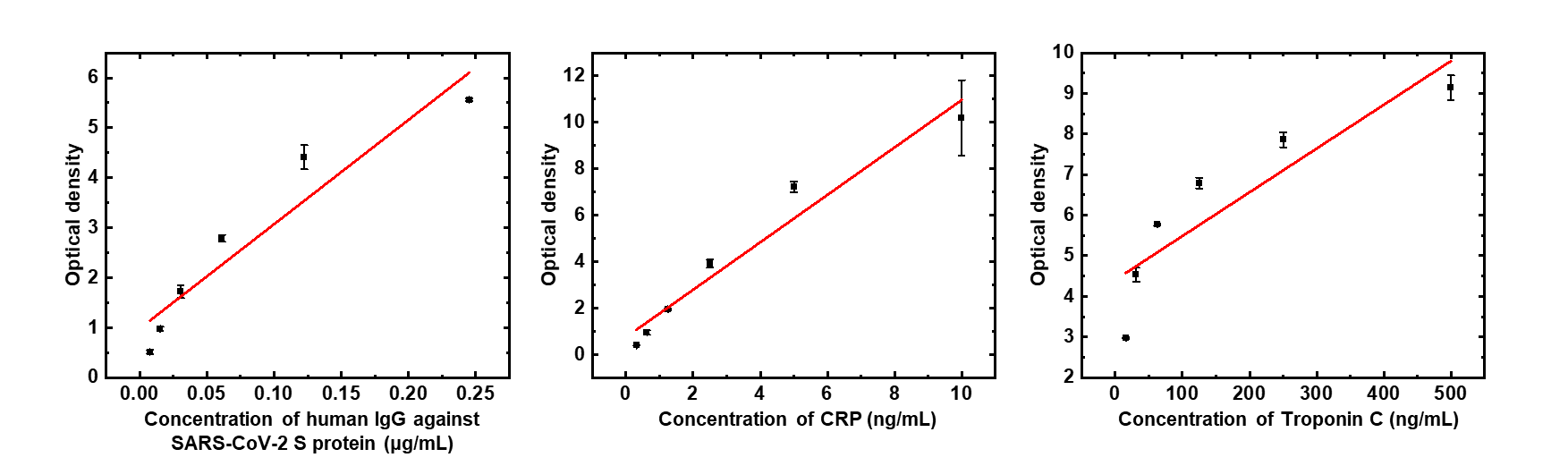


**Fig. S7: Benchmark ELISA of three targets performed in microwell plate**.

**Tab. S3: Limit of detection**.

| **Target** | **Droplab platform** | **Wellplate** |
| --- | --- | --- |
| **human IgG against SARS-CoV-2 S protein (μg/mL)** | 0.091 | 0.087 |
| **CRP (ng/mL)** | 1.611 | 2.469 |
| **Troponin (ng/mL)** | 235.561 | 284.205 |

**Supplementary Methods**

***Surface coating method of the DropLab chip***

To have a uniform coating on the surface of the recessed internal patterns, the DropLab chip was placed on an electric turntable that rotate at a constant speed during spray coating. The sprayer was tilted slightly and the nozzle was positioned 20 cm from the chip surface. The sprayer swirled about the center of the chip for 40 seconds to achieve a uniform coating.

***Preparation of microwell plate-based ELISA***

For human IgG against SARS-CoV-2 spike protein ELISA, the ELISA plate was coated with 50 μL of SARS-CoV-2 spike protein at 6.25 μg/mL in CBS at room temperature for 2 hours. After incubation, the solution was aspirated from each well, and the well was washed with 300 μL of washing buffer for 3 times and blocked with 300 μL of blocking buffer at room temperature for 1 hour. After blocking, the solution was aspirated from each well and washed with 300 μL of washing buffer for 3 times. All washing solution was removed before sample addition. 50 μL of human IgG sample against SARS-CoV-2 S protein was added to each well. After incubating at room temperature for 15 mins, the plate was washed for 3 times with 300 μL of washing buffer for 3 times. All washing solution was removed before adding the detector antibody. 50 μL of 20 ng/mL HRP-conjugated rabbit anti-human IgG for 15 minutes at room temperature. After incubation the plate was washed for 3 times with 300 μL of washing buffer for 3 times. All washing solution was removed before adding TMB. The plate was incubated with 50 μL of TMB for 15 minutes, and 50 μL of stop solution was added to each well after incubation. The results were taken by measuring the absorbance of developed TMB at 450 nm with a UV-Vis photospectrometer.

ELISA for CRP and TnC in microwell plate were done in a similar way. The concentration of the capture antibody and detector antibody was 2 μg/mL and 0.1 μg/mL, respectively.

***Measurement of microbeads retention rate***

Microbeads that remained in the last droplet (microwell 8) were collected and dried in an oven (LICHEN). 20 sets of dried beads from the last droplets in each reaction were combined and weighed using an electronic weighing scale with a weighing resolution of 0.01 mg (CNSHP). Residual microbeads in rest of the droplets (microwell 1~7 and 9) were also collected, combined and dried in the oven, and 20 sets of these samples were combined and weighed in the same way The retention rate of microbeads was calculated by comparing the amount of microbeads in microwell 8 and the total amount of microbeads (beads in microwell 8 + residual beads in the rest microwells).

**Supplementary Videos**

Video S1 Droplet moving and merging on DropLab

Video S2 extraction

Video S3 mixing

Video S4 ELISA process
